# Retraction Note: Impact of gervital against histopathological, ultrastructural, and biochemical alterations caused by methotrexate or azathioprine in albino rat testis

**DOI:** 10.1007/s11356-024-33995-3

**Published:** 2024-06-13

**Authors:** Manal Abdul-Hamid, Eman S. Abdel-Reheim, Walaa Hegazy, Ahmed A. Allam, Sarah I. Othman, Haifa ALqhtani, Samraa H. Abdel-Kawi

**Affiliations:** 1 Zoology Department, Faculty of Science, Histology and Cell Biology Division, Beni-Suef University, Beni-Suef, Egypt; 2 Zoology Department, Faculty of Science, Molecular Physiology Division, Beni-Suef University, Beni-Suef, Egypt; 3 Basic Science Department, Faculty of Physical Therapy, Histology Division, Nahda University, Beni-Suef, Egypt; 4 Department of Zoology, Faculty of Science, Beni-Suef University, Beni-Suef, Egypt; 5 Department of Biology, College of Science, Princess Nourah Bint Abdulrahman University, P.O. Box 84428, 11671 Riyadh, Saudi Arabia; 6 Medical Histology & Cell Biology Department, Faculty of Medicine, Beni-Suef University, Beni-Suef, Egypt

**Retraction Note: Environmental Science and Pollution Research (2022) 30:21914-21926**

10.1007/s11356-022-23588-3

The Editor-in-Chief has retracted this article. After publication, concerns were raised regarding areas of high similarity within the images presented in Figs. 5b and 9d. The authors have provided the raw data images for validation. However, further checks by the Publisher have found that the images presented in Figs. 4a, 5a, 5b and 9d appear to be different from the raw data provided and show signs of image editing.

The Editor-in-Chief therefore no longer has confidence in the presented data.

None of the authors have responded to any correspondence from this publisher about this retraction notice.

